# Tau K321/K353 pseudoacetylation within KXGS motifs regulates tau–microtubule interactions and inhibits aggregation

**DOI:** 10.1038/s41598-021-96627-7

**Published:** 2021-08-23

**Authors:** Yuxing Xia, Brach M. Bell, Benoit I. Giasson

**Affiliations:** 1 Department of Neuroscience, College of Medicine, University of Florida, Gainesville, FL 32610 USA; 2 Center for Translational Research in Neurodegenerative Disease, College of Medicine, University of Florida, BMS J483/CTRND, 1275 Center Drive, Gainesville, FL 32610 USA; 3 McKnight Brain Institute, College of Medicine, University of Florida, Gainesville, FL 32610 USA

**Keywords:** Neuroscience, Diseases, Neurology

## Abstract

Alzheimer’s disease is the leading cause of dementia and a defining hallmark is the progressive brain deposition of tau aggregates. The insidious accumulation of brain tau inclusions is also involved in a group of neurodegenerative diseases termed frontotemporal dementias. In all of these disorders, tau aggregates are enriched in post-translational modifications including acetylation, which has recently been identified at multiple sites. While most evidence suggest that tau acetylation is detrimental and promotes tau aggregation, a few studies support that tau acetylation within the KXGS motif can be protective and inhibit tau aggregation. To model site-specific acetylation at K259, K290, K321, and K353, acetylmimetics were created by mutating lysine to glutamine residues, which approximates size and charge of acetylation. HEK293T cells were transfected to express wild type tau, tau pathogenic mutations (P301L and P301L/S320F) or tau acetylmimetics and assessed by cell-based assays for microtubule binding and tau aggregation. Acetylmimetics within the KXGS motif (K259Q, K290Q, K321Q, K353Q) leads to significant decreased tau–microtubule interactions. Acetylmimetics K321Q and K353Q within the context of the pathogenic P301L tau mutation strongly inhibited prion-like seeded aggregation. This protective effect was confirmed to decrease intrinsic aggregation of P301L/S320F tau double mutation. Surprisingly, K321Q and K353Q acetylmimetics altered the conformational structure of P301L/S320F tau to extensively impair Thioflavin S binding. Site-specific acetylation of tau at K321 and K353 could represent a natural protective mechanism against tau aggregation and could be a potential therapeutic target.

## Introduction

Brain pathological tau inclusions are one of the major defining hallmark in Alzheimer’s disease (AD), frontotemporal dementia (FTD), progressive supranuclear palsy (PSP), corticobasal degeneration (CBD), and other neurodegenerative disorders called tauopathies^1^. Tau inclusions can be post-translationally modified and are often found to be hyperphosphorylated in late-stage disease^2,3^. In addition to phosphorylation, tau acetylation has emerged as another prominent post-translational modification^3–5^. Enzymes with acetylation activity including p300 acetyltransferase and acetyltransferase Creb-binding protein (CBP) have been identified to acetylate full length tau protein in vitro^5,6^. Additionally, tau also contains auto-acetylation activity mediated by catalytic cysteine residues C291 and C322^7^.

In AD brains, insoluble tau aggregates are enriched by acetylation at multiple sites^3^. Antibodies against acetylated K280 can detect tau pathology in different tauopathies such as AD, FTD, PSP, and CBD, which includes both familial and sporadic cases^8,9^. Likewise, acetylated K274 can label tau aggregates in AD, FTD, PSP, and CBD brains, but not argyrophilic grain disease (AGD)^10^. Tau acetylation at K174, K274, and K281 have been demonstrated to inhibit synaptic transmission and promote memory deficits in vivo^11,12^. Tau acetylation can also regulate its degradation with acetylation of K274 and K281 selectively targeting macroautophagy, while increased pseudoacetylation of these sites exacerbate pathogenesis in some disease models^13^. Overall reduction of tau acetylation also improve outcomes in models of traumatic brain injury^14^.

**Figure 1:**
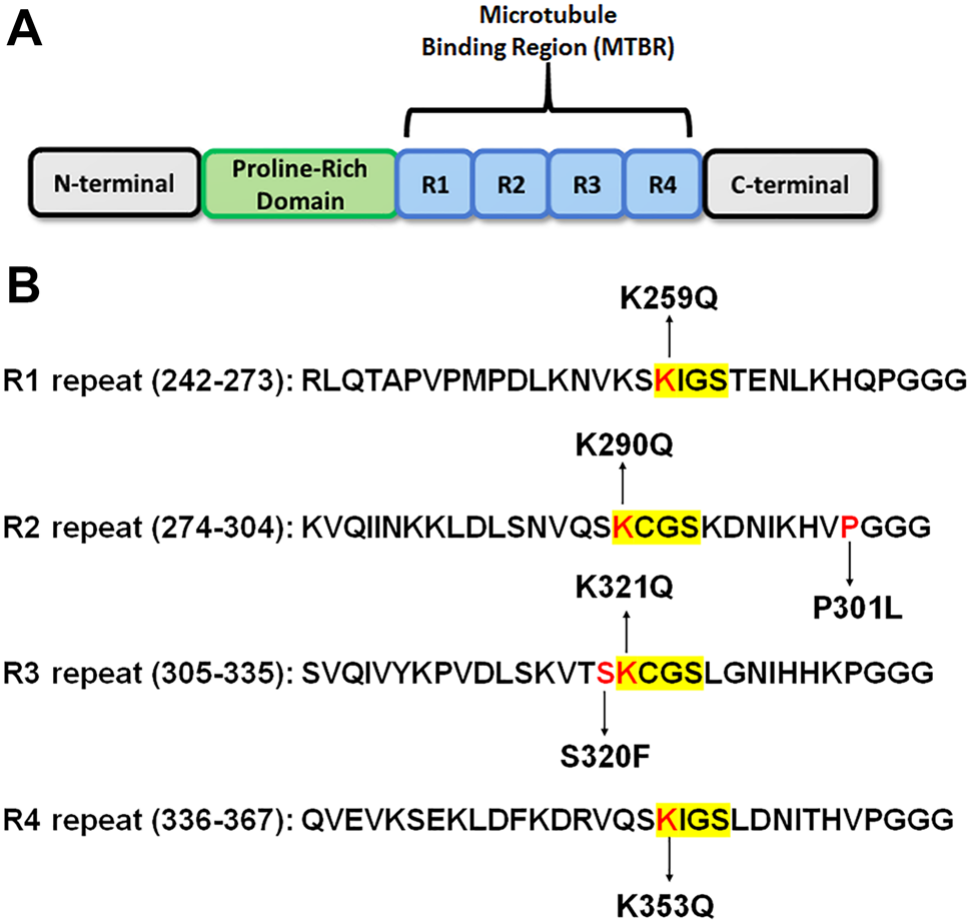
Tau protein structure and overview of pseudoacetylation and mutation sites. (**A**) Illustration shows the general structure of the tau protein with major domains: N-terminal domain, proline-rich region, MT-binding region consisting of four major repeats, and C-terminal domain. (**B**) Amino acid sequences of four MT binding repeats R1 to R4 are shown. Acetylation sites K259, K290, K321, and K353 are within conserved KCGS or KIGS motifs in each of the four major repeats, respectively, and numbered according to the 2N4R human tau isoform. Additionally, P301L and S320F MAPT missense mutations that are depicted were used as a model of intrinsic tau aggregation.

While most evidence suggests tau acetylation contributes to disease, several studies provided evidence that specific acetylation sites within the KXGS motifs (K259, K290, K321, K353) could be protective^15,16^. In rTg4510 tau transgenic mice and in AD brains, tau acetylation at KXGS motifs are decreased and may help inhibit tau phosphorylation^15^ . The KXGS motifs are well-conserved residues located in each of the four microtubule (MT)-binding repeats of tau (Fig. 1). Acetylation of KXGS motifs can be mediated by p300 acetyltransferase^5,16^ and deacetylated by histone deacetylase 6 (HDAC6)^15^.

One method to model site specific acetylation is to use pseudoacetylation mutations that substitute lysine for glutamine, which approximates the size and charge of acetylated tau^4,13–17^. In vitro studies indicate that quadruple acetylmimetic mutations at K259Q/K290Q/K321Q/K353Q are resistant to tau filament assembly^15^. Tau with either K290Q or K321Q acetylmimetics are less likely to aggregate in vitro^16^. However, it is unknown if these acetylation sites could regulate MT binding or if individual sites are sufficient to inhibit both intrinsic and prion-like seeded aggregation in a cellular context.

In this study, we expressed tau with acetylmimetics K259Q, K290Q, K321Q, and K353Q in HEK293T cells and assessed the effects on the modulation of tau-MT interactions in a cell-based MT binding assay. All four of these sites significantly decreased MT binding and we determined that K321Q and K353Q acetylmimetics can inhibit prion-like seeded aggregation in the context of the P301L tau mutation. This protective effect also decreased intrinsic aggregation of P301L/S320F double tau mutations, indicating that K321Q and K353Q are overall protective against tau aggregation.

## Results

### Tau acetylmimetics modulate tau-MT interactions

**Figure 2:**
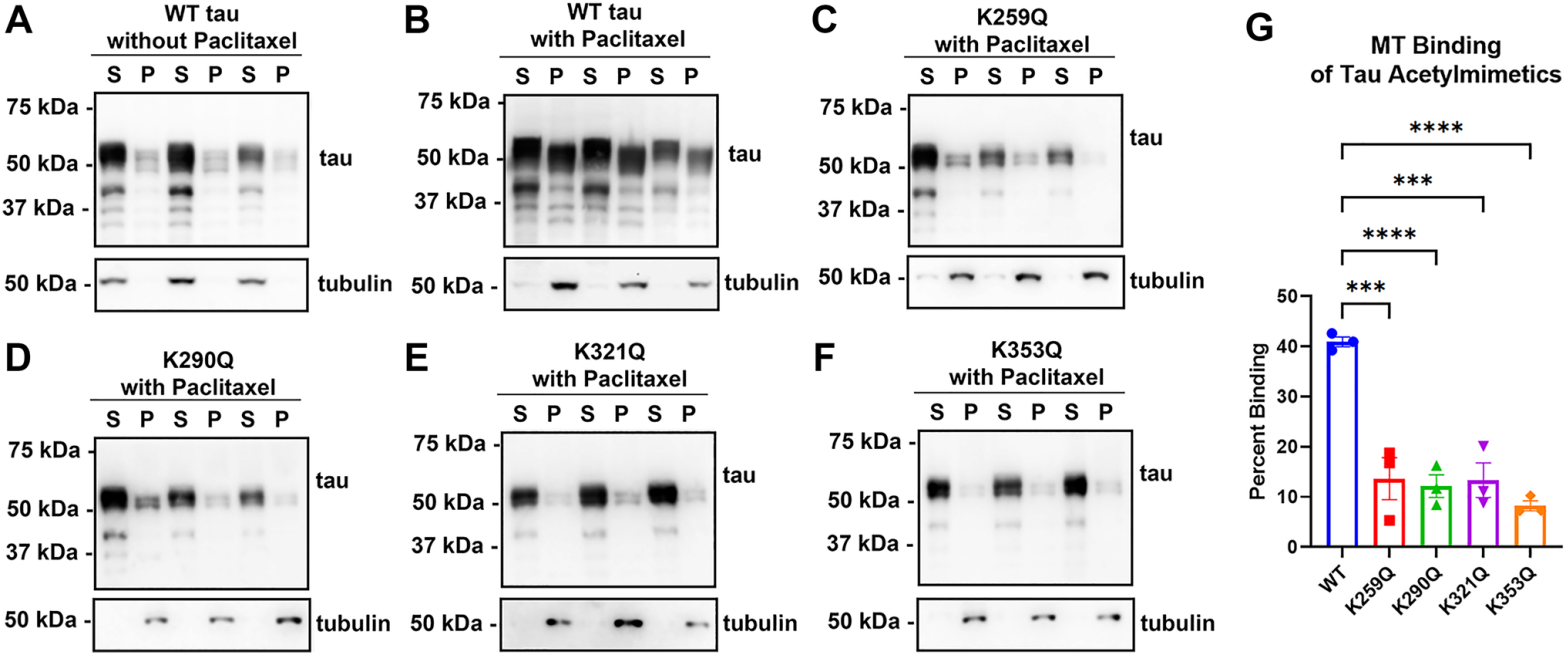
Pseudoacetylation of KXGS motif impairs MT binding. (**A,B**) A cell-based MT binding assay performed on lysate from HEK293T cells transfected with 0N4R WT tau or (**C–F**) 0N4R tau acetylation mimetics. Without Paclitaxel, most of the tubulin is unpolymerized and in the soluble fraction (**A**). With Paclitaxel, tubulin monomers polymerize as MTs in the pellet fraction (**B**). In the presence of Paclitaxel, tau acetylation mimetics (**C**) K259Q, (**D**) K290Q, (**E**) K321Q, and (**F**) K353Q lead to significant impairment of MT binding compared to WT tau. Immunoblots with antibody specific for β-tubulin (clone TUB 2.1) used to track tubulin polymerization or 3026 antibody specific for total tau. *S*  supernatants; *P*  pellet fractions. The relative molecular weight markers are shown on the left. (**G**) One-way ANOVA with Dunnett’s test was performed with N = 3 for each group. ****p < 0.0001.

Previous studies reported that acetylmimetics of multiple different sites can reduce MT bundling or impair MT binding^6,17^. Here we wanted to systematically assess the impact of acetylation sites (K259, K290, K321, and K353) within the KXGS motifs that are located within the MT-binding region (Fig. 1). These tau amino acid residues are numbered according to the 2N4R 441 amino acid human tau isoform, which is the most common nomenclature although we used the 0N4R 383 amino acid isoform for all the studies. MT binding of tau acetylmimetics compared to wild type (WT) human tau was assessed using a cell-based MT cosedimentation assay, which has been previously used to investigate the effects of tau pathogenic mutations and phosphomimetics^18,19^. In this assay, Paclitaxel can be added to cell lysates to promote tubulin polymerization into MTs. Without Paclitaxel, both tubulin and WT tau are mostly found in the soluble fraction (Fig. 2A). After adding Paclitaxel, soluble tubulin polymerizes into MTs that can be isolated in the pellet fraction after centrifugation. As a baseline, up to ~ 40% of WT tau can be found in the pellet fraction in the presence of Paclitaxel (Fig. 2B,F). Surprisingly, acetylmimetics K259Q, K290Q, K321Q, and K353Q all significantly decreased tau-MT interactions to around ~ 15% (Fig. 2C–G). This finding suggests that acetylation of any KXGS motifs can be involved in regulate tau MT binding. In this assay, full length tau in the supernatant fractions is likely more phosphorylated as it runs slightly slower on SDS-PAGE than tau in the MT-bound fractions. It is well established that higher tau phosphorylation levels are associated with reduced MT binding^20,21^ and tau more highly phosphorylated reduced its mobility on SDS-PAGE^2,22–24^.

### Tau acetylmimetics K321Q and K353Q are aggregation-resistant and inhibit prion-like seeded P301L aggregation

**Figure 3:**
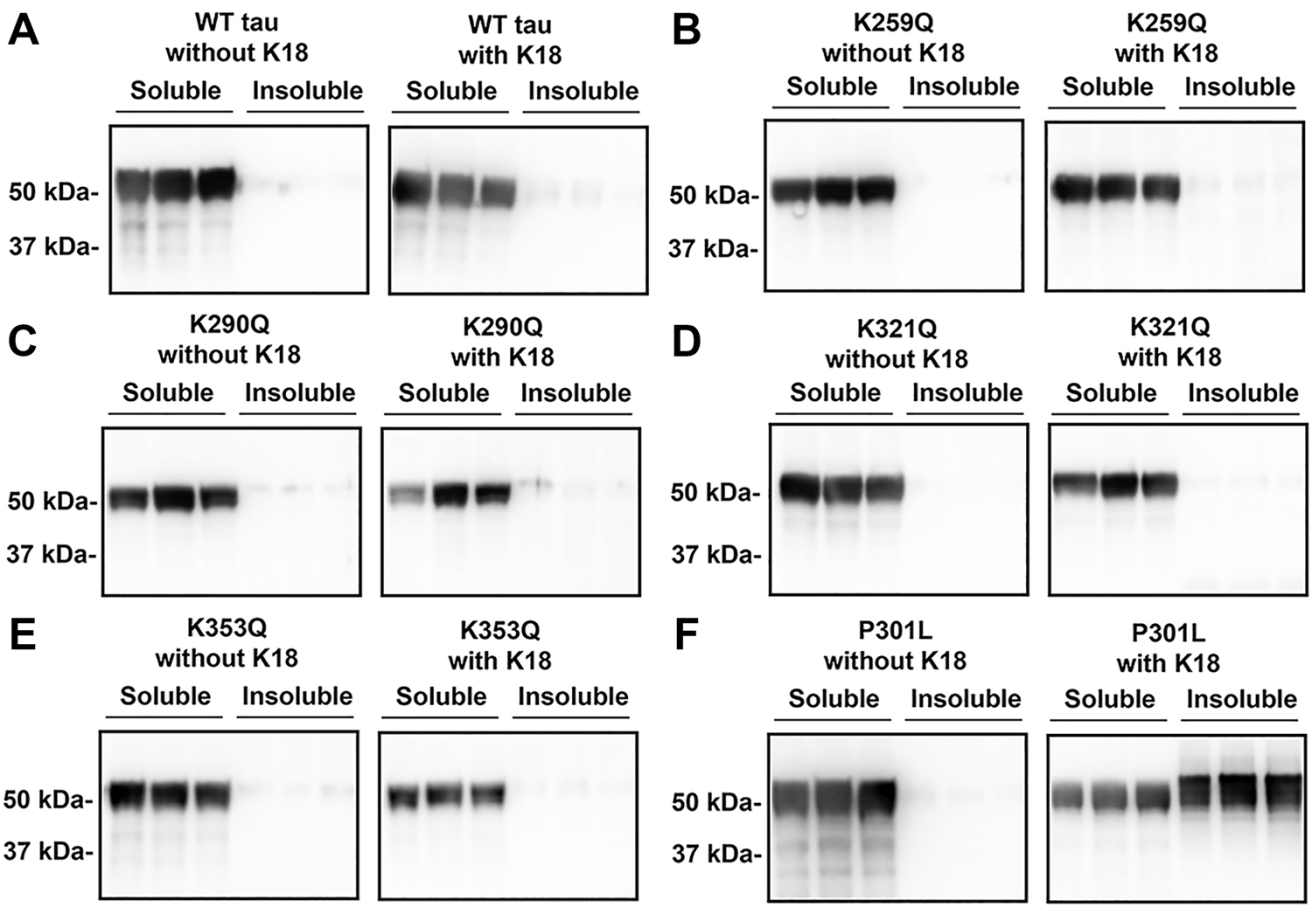
Pseudoacetylation at KXGS motifs do not lead to significant aggregation even with K18 seeds. HEK293T cells were transfected with 0N4R WT tau and 0N4R tau acetylmimetics K259Q, K290Q, K321Q, and K353Q. Cell lysates were fractionated into Triton soluble and insoluble fractions to isolate potential tau aggregates. Immunoblots were probed with tau antibody 3026. (**A**) WT tau does not aggregate with or without K18 seeds. (**B**) K259Q, (**C**) K290Q, (**D**) K321Q, and (**E**) K353Q 0N4R tau are also resistant to aggregation with or without K18 seeding. (**F**) P301L 0N4R tau was used as a positive control. The relative molecular weight markers are shown on the left.

Previous in vitro experiments showed that tau with quadruple acetylmimetics of the KXGS motifs (K259Q/K290Q/K321Q/K353Q) can decrease tau fibrillization^15^. Specifically, K290Q and K321Q decreases in vitro tau filament formation induced by dextran sulfate^16^. To test these sites individually in a cellular context, tau acetylmimetics were expressed in HEK293T cells and tau aggregation measured in a cell-based tau inclusion assay. WT tau and tau acetylmimetics K259Q, K290Q, K321Q, and K353Q were expressed in HEK293T cells and fractionated into Triton-soluble and Triton-insoluble fractions (Fig. 3). Without seeding, all four tau acetylmimetics did not significantly aggregate (Fig. 3). Even upon the addition of K18 seeds, none of the acetylmimetics showed aggregates within the Triton-insoluble fraction, suggesting that they do not directly promote aggregation as compared to tau with the P301L mutation that can be readily induced to aggregate in the presence of K18 seeds (Fig. 3F)^19,25,26^ and that is associated with familial FTD^27,28^. In this assay full length tau in the insoluble fractions display a slight shift to higher apparent molecular weight as it run slower on SDS-PAGE than tau in the soluble fractions. It is well established that increased tau phosphorylation is prone to aggregation^29–32^ and highly phosphorylated tau has reduced mobility on SDS-PAGE^2,22–24^.

**Figure 4:**
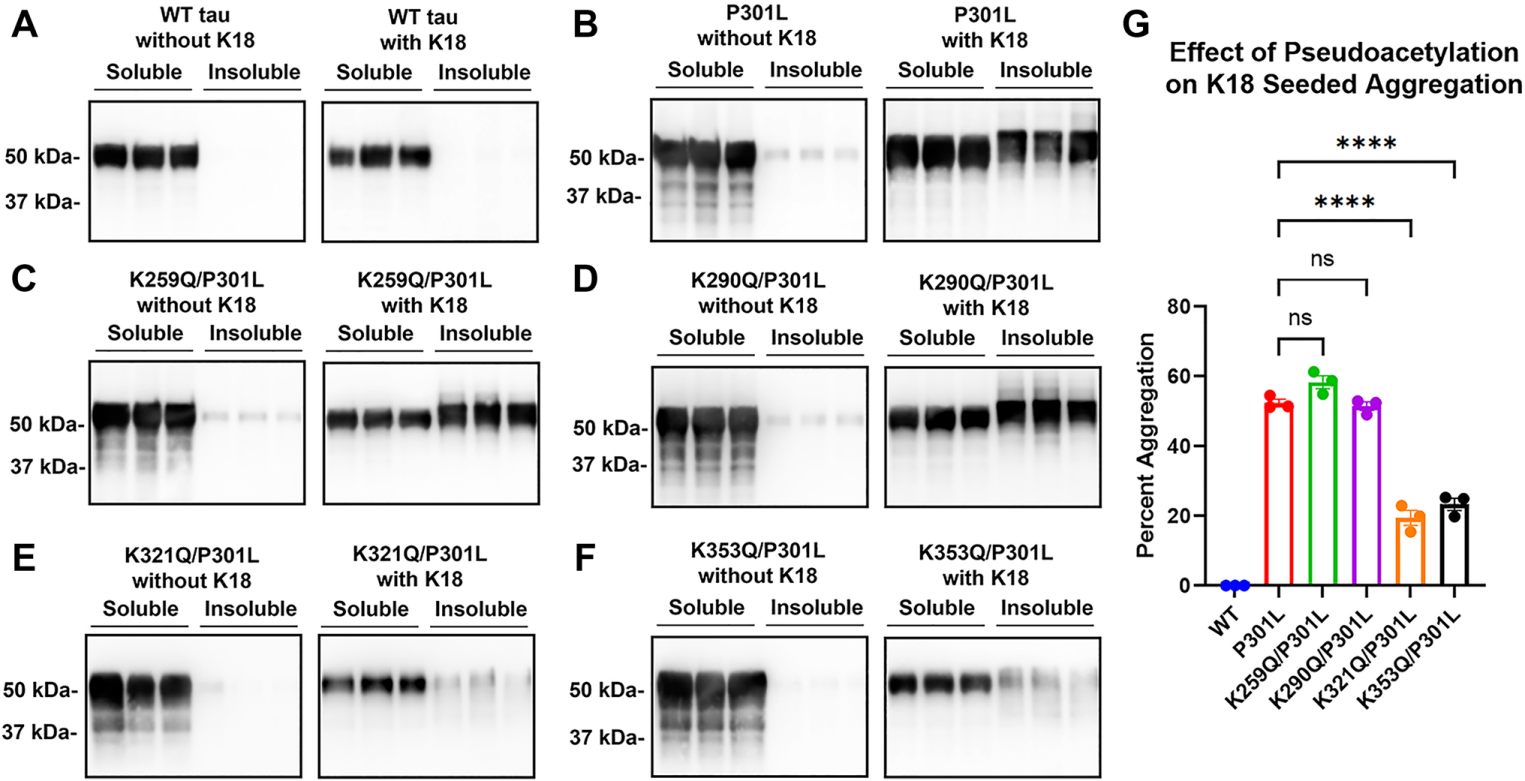
K321Q and K353Q tau acetylmimetics inhibit prion-like seeded aggregation in the context of P301L mutation. Cell-based tau inclusion assays were used to determine aggregation and prion-like seeding of (**A**) 0N4R WT tau, and (**B**) P301L, (**C**) K259Q/P301L, (**D**) K290Q/P301L, (**E**) K321Q/P301L/, and (**F**) K353Q/ P301L 0N4R tau. Immunoblots were probed with tau antibody 3026. The relative molecular weight markers are shown on the left. (**G**) One-way ANOVA with Dunnett’s test was performed with N = 3 for WT tau and all mutations. ****p < 0.0001 and *ns* not statistically significant.

Next, we evaluated whether acetylmimetics can modulate prion-like seeded aggregation in the context of the tau mutation P301L. K259Q, K290Q, K321Q, and K353Q acetylmimetics were combined with P301L and seeding was induced with K18 tau preformed fibrils. WT tau did not significantly aggregate with or without K18 seeding (Fig. 4A). By contrast, P301L robustly aggregated after K18 seeding (Fig. 4B). Compared with P301L, K259Q/P301L and K290Q/P301L mutations did not present with significantly altered seeded aggregation (Fig. 4C,D,G). However, K321Q/P301L and K353Q/P301L tau displayed significant resistance to tau aggregation with > 50% reduction in Triton-insoluble tau compared to P301L tau (Fig. 4E–G) in the presence of exogenous K18 tau preformed fibrils. Hence, K321Q and K353Q acetylmimetics are protective against prion-like seeded aggregation, even in the context of a pro-aggregant mutation like P301L.

### Tau acetylmimetics K321Q and K353Q inhibit intrinsic aggregation and alter conformational amyloid structure of P301L/S320F tau aggregates

**Figure 5:**
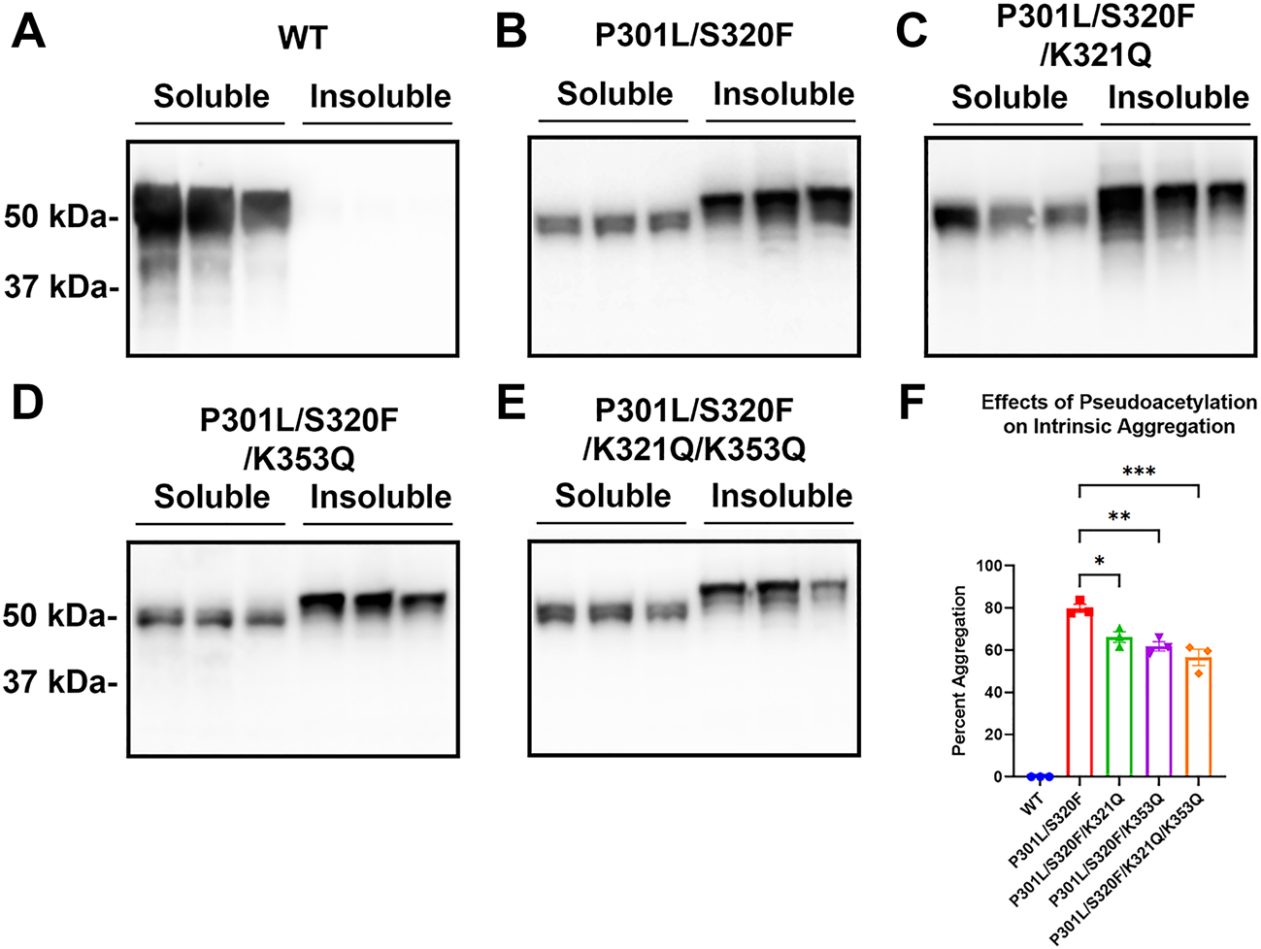
Acetylmimetics K321Q and K353Q inhibit self-aggregation of P301L/S320F tau double mutants. Cell-based tau inclusion assays were used to assess intrinsic aggregation of (**A**) WT 0N4R tau, or 0N4R tau mutants (**B**) P301L/S320F, (**C**) P301L/S320F/K321Q, (**D**) P301L/S320F/K353Q, and (**E**) P301L/S320F/K321Q/K353Q. Immunoblots were probed with tau antibody 3026. The relative molecular weight markers are shown on the left. (**F**) Graph shows percent aggregation of WT tau and different tau mutations. One-way ANOVA with Dunnett’s Test was performed with N = 3 for WT tau and all mutations. *p < 0.05, **p < 0.01, and ***p < 0.001.

Since K321Q and K353Q acetylmimetics may be protective against seeded tau aggregation, these acetylmimetics were also tested in a model of intrinsic aggregation. Our lab has previously discovered that P301L/S320F double mutation robustly aggregates without seeding^19,26^. K321Q and K353Q were combined with P301L/S320F tau to assess whether these acetylmimetics can also inhibit intrinsic aggregation. Compared to WT tau, P301L/S320F aggregates up to ~ 80% without seeding (Fig. 5A,B,F). Both P301L/S320F/K321Q and P301L/S320F/K353Q had decreased aggregation with levels ~ 60%, showing a relative 25% reduction (Fig. 5C,D,F). K321Q and K353Q were combined with P301L/S320F (P301L/S320F/K321Q/K353Q), which had aggregation levels comparable to P301L/S320F/K321Q and P301L/S320F/ K353Q (Fig. 5E,F). While K321Q and K353Q acetylmimetics both protect against intrinsic aggregation, they do not appear to have an additive effect.

**Figure 6:**
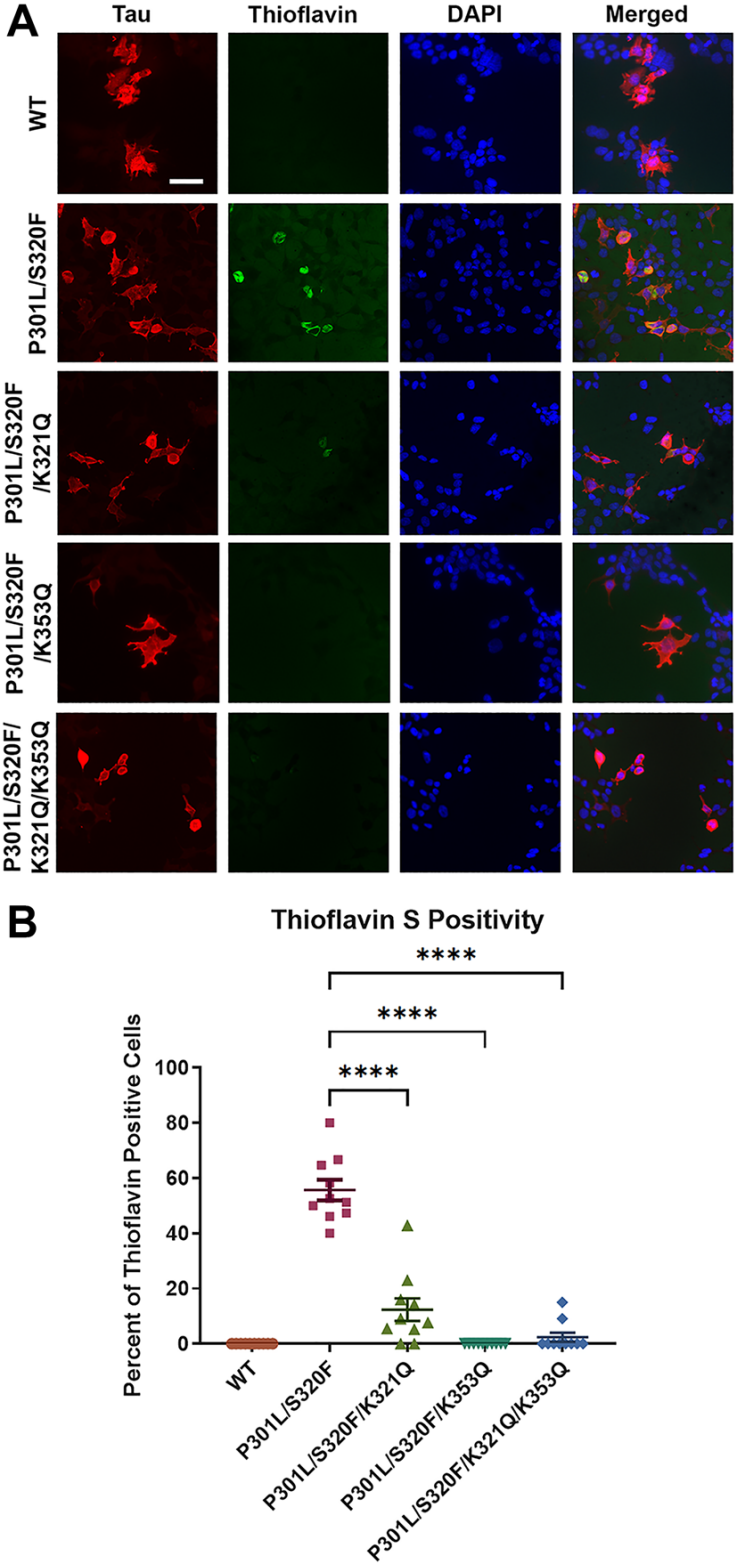
In the context of the P301L/S320F aggregation mutations, K321Q and K353Q acetylmimetics disrupt conformational amyloid structure as shown by reduced Thioflavin reactivity. **(A)** HEK293T cells were transfected to express WT, P301L/S320F, P301L/S320F/K321Q, P301L/S320F/K353Q, or P301L/S320F/K321Q/K353Q 0N4R tau. Cells were plated onto slides and labeled for fluorescence with DAPI for nuclei, 3026 antibody/Alexa 594 for total tau (red), and Thioflavin S for amyloid structure (green). (**B**) Graph shows the ratio of thioflavin positive to tau positive cells calculated from 10 different ×20 fields for each group. One-way ANOVA with Dunnett’s test was performed with N = 10 for WT tau and mutations. ****p < 0.0001. Scale bar = 50 µm.

To directly visualize tau aggregates, cells transfected to express these various acetylmimetics in the context of P301L/S320F were stained with Thioflavin S, which is a fluorescent dye that strongly binds beta-pleated sheets of amyloid structures^33,34^ (Fig. 6, Supplemental Fig. 2). Since WT tau did not form aggregates, green Thioflavin S signal was not detected in cells transfected with WT tau. Approximately 60% of cells transfected to express P301L/S320F tau contained Thioflavin positive aggregates (Fig. 6A,B). Surprisingly, combining K321Q with P301L/S320F tau mutants decreased Thioflavin positivity to ~ 15% (Fig. 6A,B). Almost no Thioflavin signal was found in cells expressing P301L/S320F/K353Q and P301L/S320F/K321Q/K353Q tau (Fig. 6, Supplemental Fig. 2). This finding suggests that K321Q and K353Q can alter the conformational amyloid structure of P301L/S320F tau aggregates and prevent Thioflavin S dye from binding, which were more pronounced than the differences in biochemical aggregation (Fig. 5).

## Discussion

Tau acetylation has been recently confirmed to be a major post-translational modification and is elevated in tauopathies^3–5^. However, different studies suggest that tau acetylation can both promote and inhibit tau aggregation depending on the modified site. Thus studies of overall tau acetylation are complicated by the offsetting impact of simultaneous acetylation of different amino acid residues. One approach to investigate the role of site specific acetylation is to use pseudoacetylation mutations that substitute lysine for glutamine, which approximates the size and charge of acetylated tau^4,13–17^ with the caveat that this substitution is not identical to acetylated lysine. Unlike most acetylation sites, acetylation of KXGS motifs appear to be decreased in transgenic tau models and in post-mortem tissue from AD patients^16^. In this region, acetylmimetics K290Q and K321Q have been previously shown to inhibit tau aggregation in vitro^16^. Using cell-based assays in transfected HEK293T cells, we examined MT binding and aggregation of tau acetylmimetics at the conserved KXGS motifs (K259, K290, K321, and K353). All four of these sites are located within the MT-binding region in each of the four repeats (Fig. 1). Tau acetylmimetics at any of these sites displayed decreased tau-MT interactions by > 50% relative to WT to levels comparable to the effects of many pathogenic tau mutations^19^. This type of effect on tau-MT interaction was also found for some phosphomimetics like S262^20,21^, implying that both acetylation and phosphorylation are involved in regulation of MTs. Decreased MT binding of tau may indirectly facilitate protein degradation while tau acetylation of K274, K290, K321 and K353 is also involved in regulating tau chaperone interactions and tau degradation^35^ that could contribute to the removal of pathogenic forms of tau.

Tau acetylmimetics at K321 and K353, but not K259 and K290, inhibited prion-like aggregation of P301L tau mutant seeded with exogenous K18 tau preformed fibrils. This protective effect was confirmed to a lesser extent in the context of P301L/S320F tau double mutant, which robustly aggregates without seeding^19,26^. Based on Cryo-EM studies, R3 and R4 repeats of tau (306–378 residues) form the core of tau aggregates^36^. Both K321 and K353 are potential acetylation sites within this region and may directly affect tau filament formation.

Our data indicate that K321Q and K353Q significantly altered the conformational structure of mutant tau (P301L/S320F) as their presence almost completely prevented the binding of fluorescent Thioflavin S dye, which is specific for amyloid structure of protein aggregates^33,34^. This is unexpected since these tau aggregates are still detergent insoluble despite being Thioflavin negative. The actual protein structure and sequence required for Thioflavin S binding is still not well defined although Thioflavin S is routinely used as a marker of protein amyloid formation. However, a similar effect was also found in K280 acetylmimetic in P301S transgenic mice where K280Q significantly decreased tau filament formation and Thioflavin S reactivity^37^. Tau acetylation within the MT-binding region likely interferes with packing of beta-pleated sheets involved in amyloid formation and thus either tertiary or/and quaternary structures resulting in polymorphs, but that are still permissive to protein inclusion formation. The inhibition of Thioflavin S reactivity due to the K321Q and K353Q amino acid substitutions is also akin to structure prion-like conformation changes. For example, it was recently shown that during fibrillization of α-synuclein Thioflavin-negative polymorphs can arise and that these can be transmitted with prion-like properties that are maintained during passaging^38^.

One limitation of this study is that tau mutations were used to model aggregation and prion-like seeding, and most patients with tauopathies do not have tau missense mutations. However, these findings can be used as a model of how tau aggregation could potentially be inhibited if WT tau adopts similar pathogenic conformation in patients with sporadic tauopathies.

Overall, pseudoacetylation of KXGS motifs have protective effects against tau aggregation. Specifically, K321Q and K353Q strongly inhibited both intrinsic and prion-like seeded aggregation of different tau mutations. However, this effect did not appear to be additive, which suggests single acetylation sites at either K321 or K353 could be sufficient to prevent or slow the formation of tau filaments. Paradoxically, increased pseudoacetylation of these same sites also decreased MT binding, which is thought to be a detrimental effect associated with many tau missense mutations^19,39^. Pseudoacetylation is a model of constitutive acetylation and physiologic baseline levels of tau acetylation may be low enough that normally MTs are minimally affected. In addition, acetylation is a reversible modification so that it can be involved in regulating MT-tau interaction. Transient hyper-acetylation could be used to temporally inhibit tau aggregation, while the lost of tau-MT interaction could be reserved by subsequent deacetylation. When tau aggregates begin to form, tau acetylation of specific sites like K321 and K353 may be elevated as a compensatory response to inhibit additional tau aggregation and would also facilitate and promote proteasomal degradation of these tau aggregates^35^. Future studies on tau acetylation could better elucidate the complex relationships between tau acetylation and its effects on MTs and tau aggregation.

## Methods

### Protein purification of K18 tau and fibrillization

K18 amyloid tau seeds were generated from the tau protein fragment K18, which contains the MT binding repeats from Q244 to E372 (numbering based on 2N4R full length tau) as previously described^19,26,39^.

### Plasmid cloning and site-directed mutagenesis

The 0N4R human tau cDNA isoform was cloned into mammalian expression vector pcDNA3.1 ( +). Tau acetylmimetics were created with QuikChange site-directed mutagenesis (Agilent Technologies, Santa Clara, CA) using partially overlapping oligonucleotides^40^. Correct tau sequences were verified and sequenced by Genewiz (South Plainfield, NJ).

### Cell culture and calcium phosphate transfection

HEK293T cells were grown in Dulbecco’s modified eagle media with 10% fetal bovine serum (FBS) and antibiotics (100 U/ml penicillin, 100 µg/ml streptomycin) at 37 ºC and 5% CO_2_. Cell transfection was performed by using calcium phosphate precipitation as previously described^19,41^. For cell seeding studies, 1 µM of K18 tau fibrils were added one hour after transfection as previously described^19,41^. At 16 h after transfection, cells were exchanged and maintained in media with only 3% FBS. Cells were harvested 48 h after the media change.

### Cell-based tau aggregation assay

Cell lysates were prepared in 200 µL of Triton Lysis Buffer (25 mM Tris–HCl, pH 7.5, 150 mM NaCl, 1 mM EDTA, 1% Triton X-100, 20 mM NaF) with a mix of protease inhibitors (1 mM phenylmethylsulfonyl and 1 mg/ml each of pepstatin, leupeptin, *N*-tosyl-l-phenylalanyl chloromethyl ketone, *N*-tosyl-lysine chloromethyl ketone and soybean trypsin inhibitor) like previous studies^18,19,26^. These solutions were centrifuged at 100,000 × *g* and 4 ºC for 30 min. The Triton soluble fractions were collected separately. The Triton insoluble fractions were washed with additional buffer and centrifuged again at 100,000 × *g* and 4 ºC for 30 min. After the wash step, the pellet was resuspended in Triton Lysis Buffer. SDS loading buffer (10 mM Tris, pH 6.8, 1 mM EDTA, 40 mM DTT, 0.005% bromophenol blue, 0.0025% pyronin yellow, 1% SDS, 10% sucrose) was added to both soluble and insoluble fractions and boiled for 10 min. The Triton insoluble fraction was probe-sonicated and boiled again for 10 min. Percent aggregation was calculated as a ratio of insoluble tau/(soluble tau + insoluble tau) × 100.

### Cell-based MT binding assay

Cells were lysed in 200 µL of PEM buffer (80 mM PIPES, pH 6.8, 1 mM EGTA, 1 mM MgCl_2_) supplemented with 0.1% Triton X-100, 2 mM GTP, 20 µM Paclitaxel, and a mix of protease inhibitors (1 mM phenylmethylsulfonyl and 1 mg/ml each of pepstatin, leupeptin, *N*-tosyl-l-phenylalanyl chloromethyl ketone, *N*-tosyl-lysine chloromethyl ketone and soybean trypsin inhibitor) as previously described^18,19^. Cell lysates were incubated in a 37 ºC water bath for 30 min and then centrifuged at 100,000 × *g* for 30 min to isolate MTs in the pellet. Supernatant was transferred to a new tube and the pellet (MT fraction with bound proteins) was resuspended in PEM buffer. The pellet fraction was homogenized and SDS loading buffer was added to both fractions. Equal amounts of supernatant and pellet were loaded on acrylamide gels for immunoblotting. Percent MT bound tau was calculated as pellet/(supernatant + pellet) × 100.

### Western blotting

Equal proportions of each sample were loaded on 10% polyacrylamide gels and separated by SDS-PAGE. After transfer, the membranes were blocked in 5% milk with Tris-buffered saline (TBS) for an hour. The membranes were incubated in primary antibody overnight at 4 ºC at dilutions of 1:1000 for β-tubulin and tau antibodies. Anti-β-tubulin (Clone TUB 2.1) is a mouse monoclonal antibody (Sigma-Aldrich, St. Louis, MO) and 3026 tau antibody is a rabbit polyclonal antibody that was raised against full length 0N3R human tau but also reacts with 0N4R human tau (Supplemental Fig. 1)^26,42,43^. After TBS washes, goat anti-rabbit or anti-mouse secondary antibodies conjugated to horseradish peroxidase (Jackson Immuno Research labs, Westgrove, PA) were added to the membranes at 1:4000 dilution for an hour. After several washes, the membranes were exposed and imaged after adding Western Lightning Plus ECL reagents (PerkinElmer, Waltham, MA). Each lane was semi-quantitatively measured in ImageJ by densitometric analysis. Statistical tests were performed on GraphPad Prism version 8.4.3 for one way analysis of variance (ANOVA) with post hoc analysis and Dunnett’s test.

### Immunofluorescence with thioflavin staining

HEK293T cells were rinsed in phosphate-buffered saline (PBS) and fixed in 4% paraformaldehyde for 10 min. After washing, autofluorescence eliminator reagent (Millipore, Burlington, MA) was added for five minutes and washed with 40% ethanol. Under dark conditions, slides with cells were incubated in 0.0125% Thioflavin S dissolved in 50% ethanol/PBS for 3 min. Thioflavin S was washed off with 50% ethanol and PBS. Slides were then placed in blocking solution (2% FBS/0.1% Triton-X-100 in PBS) for 30 min. Primary antibody (3026 antibody against total tau^42^) in 2% FBS/PBS was added for one hour. After PBS washes, Alexa 594-conjugated anti-rabbit secondary antibodies (1:500 dilution) (Invitrogen, Carlsbad, CA) were added for one hour. Slides were washed in PBS and stained with 0.5 µg/ml 4’,6-diamidino-2-phenylindole (DAPI, Invitrogen, Carlsbad, CA) in PBS for 5 min. After PBS washes, the slides were mounted using Fluoromount-G (Invitrogen, Carlsbad, CA). Fluorescent images were taken using a BZ-X700 Keyence digital microscope (Itasca, Il). Cell counting was performed by BMB using ImageJ. Percent Thioflavin reactivity was calculated from a ratio of Thioflavin positive to tau positive cells, as determined by the total tau antibody 3026.

## Supplementary Information

Supplementary Figures.
