# Supplementary material for: Tau K321/K353 pseudoacetylation within KXGS motifs regulates tau–microtubule interactions and inhibits aggregation
Source: Sci Rep. 2021 Aug 23;11:17069. doi: 10.1038/s41598-021-96627-7 (PMC8382713; doi:10.1038/s41598-021-96627-7)
Supplement: Supplementary file 1 — Supplementary Figures. [file 41598_2021_96627_MOESM1_ESM.docx]

**Tau K321/K353 pseudoacetylation within KXGS motifs regulate tau-microtubule interactions and inhibit aggregation**

Supplemental Information

Yuxing Xia^1,2,3^, Brach M. Bell^1,2,3^, Benoit I. Giasson^1,2,3*^

^1^Department of Neuroscience, College of Medicine, University of Florida, Gainesville, Florida 32610, USA

^2^Center for Translational Research in Neurodegenerative Disease, College of Medicine, University of Florida, Gainesville, Florida 32610, USA

^3^McKnight Brain Institute, College of Medicine, University of Florida, Gainesville, Florida 32610, USA.

**
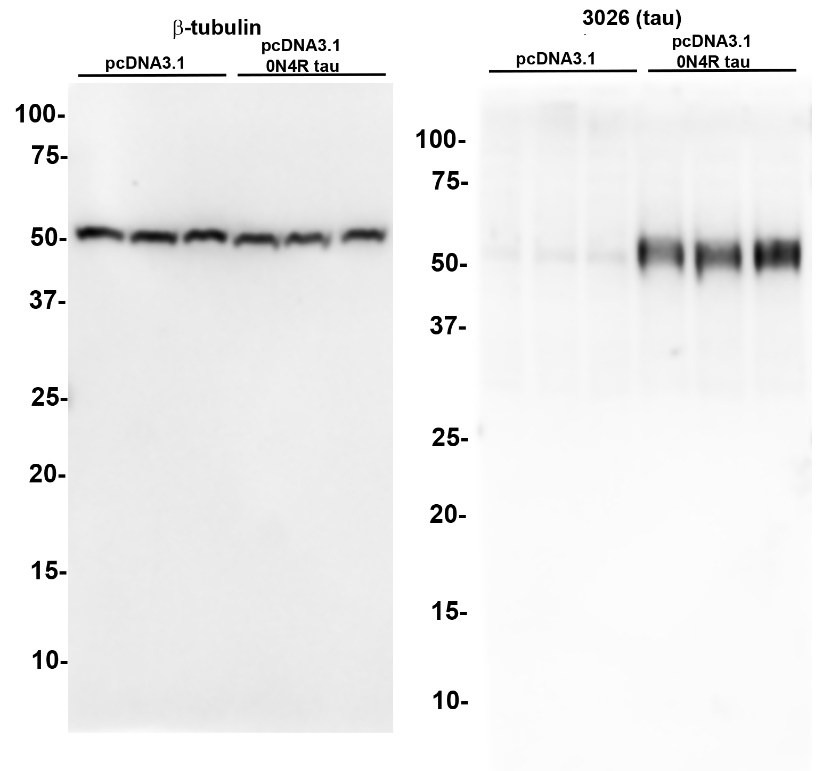
**

**Figure S1. Rabbit polyclonal antibody 3026 specifically detects tau.**

HEK293T cells were mock transfected (empty pcDNA3.1) or transfected to overexpress 0N4R human tau (pcDNA3.1-0N4R tau) in triplicate. 5 μg of total protein was load on separate lanes of SDS-polyacrylamide gel that was assess by Western Blotting with anti-β-tubulin antibody TUB2.1 or anti-tau antibody 3026. The relative molecular weight markers are shown on the left.


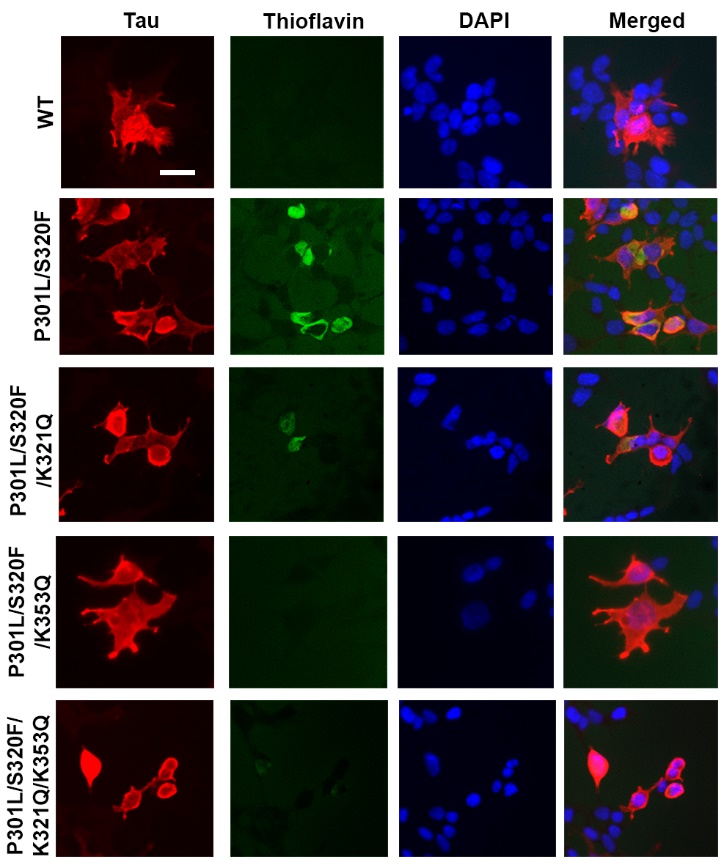


**Figure S2. Higher magnification of double immunofluorescence and Thioflavin S staining of tau acetylmimetics used in Figure 6.** HEK293T cells were transfected with WT tau and tau acetylmimetics and immunolabeled with DAPI for nuclei, 3026 antibody for total tau (red), and Thioflavin S for amyloid structure (green). Scale bar = 25 μm.
